# *In vitro* and *in vivo* anticancer efficacy of unconjugated humanised anti-CEA monoclonal antibodies

**DOI:** 10.1038/sj.bjc.6604548

**Published:** 2008-08-12

**Authors:** R D Blumenthal, H J Hansen, D M Goldenberg

**Affiliations:** 1 Garden State Cancer Center, Center for Molecular Medicine and Immunology, Belleville, New Jersey, USA; 2 Immunomedics Inc., Morris Plains, New Jersey, USA

**Sir,**

Elevated expression of carcinoembryonic antigen (CEA; CD66e; CEACAM5) has been implicated in various biological aspects of neoplasia. Carcinoembryonic antigen has been shown to be involved in both homophilic and heterophilic binding (Benchimol *et al*, 1989), suggesting to some that it is an intercellular adhesion molecule involved in cancer invasion, adhesion, and metastasis (Yoshioka *et al*, 1998). Carcinoembryonic antigen also serves an anti-apoptotic function (Ordonez *et al*, 2000). Studies have shown that CEA affects the expression of various groups of cancer-related genes, especially cell cycle and apoptotic genes, protecting colonic tumour cells from various apoptotic stimuli, including 5-FU therapy (Soeth *et al*, 2001).

Conaghan *et al*, in the March issue of *British Journal of Cancer*, described *in vitro* studies with a humanised anti-CEA antibody (PR1A3), demonstrating its ability to react with a panel of CEA-expressing colorectal cancer cell lines and induce ADCC activity. The authors state that ‘there are so far no unconjugated, or ‘naked’ antibodies to CEA being used for treatment of colorectal cancer’ (Conaghan *et al*, 2008). Since this is not correct, we offer this clarification. Dr Conaghan appears to be unaware of our work in the field of anti-CEA therapy with the MN-14 (labetuzumab) antibody. We have been studying the humanised anti-CEA monoclonal antibody (MAb), hMN-14 or labetuzimab, both prelinically and in patients, for a number of years. This MAb binds with high affinity to the restricted A3B3 domain (Gold group 3) found on CEA (Sharkey *et al*, 1995). Clinically, the MN-14 antibody was shown to have excellent targeting properties and the potential for reduced immunogenicity (Sharkey *et al*, 1995), and has been studied as a naked MAb in colorectal and breast cancer patients (Immunomedics Inc., unpublished results), as well as a radioconjugate (Hajjar *et al*, 2002; Liersch *et al*, 2005). The technology to produce large quantities of recombinant humanised MN-14 antibody in a bioreactor has been described (Qu *et al*, 2005). Furthermore, chimeric human T cells have been created that express humanised MN-14 Fab, and MN-14 scFv joined to immunoglobulin–T-cell receptors. These ‘designer T cells’ effectively kill CEA-expressing cancer cells, even in the presence of high level of soluble CEA (Nolan *et al*, 1999).

Labetuzumab, as an IgG_1_ MAb, induces effector-cell function (ADCC) *in vitro* against CEA-positive human colonic tumour cells (Blumenthal *et al*, 2005b). Antibody targeting of CEA may also modulate migration, invasion, and adhesion of human cancer cells *in vitro* (Blumenthal *et al*, 2005a). Labetuzumab is able to inhibit the growth of lung metastasis from colorectal cancer cells expressing high levels of CEA or from colorectal cancer cells with the lower expression of CEA, but in mice where levels of peripheral WBCs were elevated by G-CSF (Blumenthal *et al*, 2005b). The MAb also shows chemosensitising properties in both s.c. and metastatic human colonic tumour cells propagated in nude mice. Administration of labetuzumab enhanced the therapeutic effects of 5-FU and CPT-11, two cytotoxic drugs frequently used in colorectal cancer therapy (Blumenthal *et al*, 2005b). In another high CEA-expressing human medullary thyroid cancer xenograft (TT), we have shown that hMN-14 anti-CEA IgG can inhibit tumour cell growth and also augment the effects of dacarbazine, which is active in this cancer type (Stein and Goldenberg, 2004).

Although the PR1A3 antibody described by Conaghan *et al* in March 2008 demonstrates some promising *in vitro* activity, the experience with such humanised anti-CEA MAbs goes well beyond these observations, as described by our own *in vitro* and preclinical studies demonstrating direct, specific, anti-tumour effects without conjugation to a cytotoxic agent, in both colorectal and medullary thryroid cancer xenografts, providing evidence for the superiority of the combined modality naked anti-CEA MAb immunotherapy and chemotherapy treatments. The results support further studies focused on the integration of anti-CEA MAb therapy into chemotherapeutic regimens for the improved management CEA-expressing neoplasms.
